# Hyperparathyroidism-Jaw Tumor Syndrome Associated to a CDC73 Gene Pathogenic VARIANT and a Nonossifying Desmoplastic Fibroma of the Mandible

**DOI:** 10.1155/crie/4340464

**Published:** 2025-06-18

**Authors:** Castellano Elena, Craparo Andrea, Fabrizia Di Giovanni, Linari Alessandra, Fortunato Mirella, Maffè Antonella

**Affiliations:** ^1^Department of Endocrinology, Diabetes and Metabolism, Santa Croce and Carle Hospital, Cuneo 12100, Italy; ^2^Division of Pathology, Città della Salute Hospital, Torino 10100, Italy; ^3^Department of Pathology, Santa Croce and Carle Hospital, Cuneo 12100, Italy; ^4^Genetics and Molecular Biology Unit, Santa Croce and Carle Hospital, Cuneo 12100, Italy

**Keywords:** HPT-JT syndrome, ossifying fibroma, PHPT

## Abstract

Most cases of primary hyperparathyroidism (PHPT) are sporadic and are caused by parathyroid adenomas. Hereditary forms may occur in up to 10% of PHPT patients and are more frequent in younger patients. The hyperparathyroidism-jaw tumor (HPT-JT) syndrome is characterized by PHPT in up to 95% of patients and ossifying fibromas in the jaw in 25%–50%. We describe the case of a 35-year-old male from Bangladesh referred to our hospital due to a voluminous right mandibular swelling: a rare nonossifying fibroma of the mandible was diagnosed. Due to functional impotence, a left shoulder magnetic resonance imaging (MRI) was performed with evidence of a pluri-lobulated cyst-like lesion in the proximal humeral area diagnosed as a brown tumor (BT). Subsequent tests highlighted hypercalcemia and hypophosphatemia with high PTH levels. A heterozygous CDC73 pathogenic variant c.96>A p.Trp32Ter was identified. To the best of our knowledge, this is the first reported case of HPT-JT syndrome related to a CDC73 pathogenic variant, associated to a BT of the arm and a rare nonossifying fibroma of the mandible.

## 1. Introduction

Most cases of primary hyperparathyroidism (PHPT) [1] are sporadic and are caused by parathyroid adenomas. Hereditary forms may occur in up to 10% of PHPT patients, and their recognition is important for implementation of gene-specific screening protocols and investigations for other associated tumors [2, 3]. Hyperparathyroidism-jaw tumor (HPT-JT) syndrome is characterized by PHPT in up to 95% of patients and ossifying fibromas in the jaw in 25%–50% [3]. We describe a case of HPT-JT syndrome related to a CDC73 pathogenic variant, associated to a brown tumor (BT) of the arm and a rare nonossifying fibroma of the mandible.

## 2. Case Presentation

**Figure 1:**
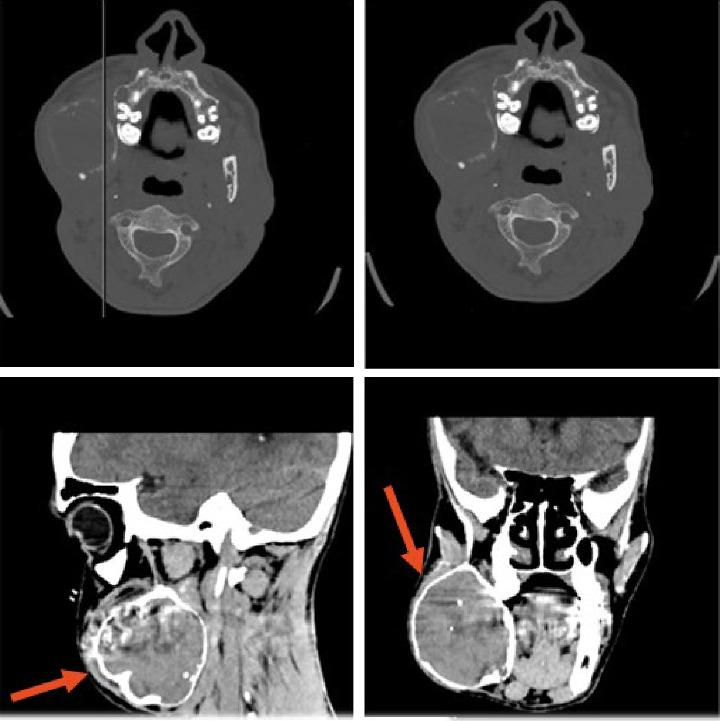
CT of the mandible. It shows an expansive lytic lesion in the right mandibular bone body.

**Figure 2:**
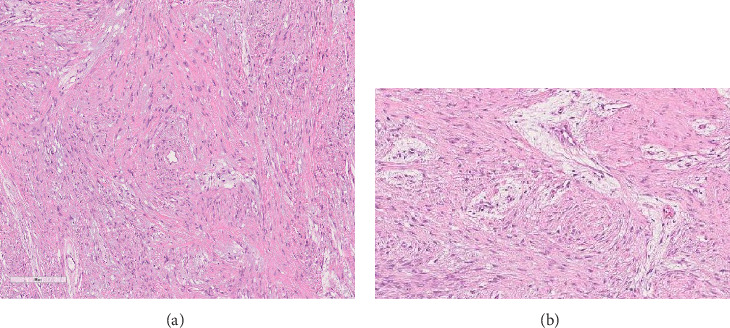
Desmoplastic fibroma of the mandible. (A) (H&E stain, 4×) Desmoplastic fibroma of the mandible. (B) At higher power, the cells have ovoid to tapering nuclei, fine chromatin and, small nucleoli, with minimal atypia (H&E stain, 10×).

A 35-year-old male from Bangladesh was referred to the maxillofacial surgery division of Santa Croce Hospital in Cuneo, Italy due to a voluminous right mandibular swelling. A computed tomography (CT) scan of the mandible showed an expansive lytic lesion on the right mandibular bone (6.1 cm × 5.3 cm × 6.7 cm), referable in the first hypothesis to ameloblastoma (Figure 1). He underwent excision of the lesion of the right hemimandible, then osteosynthesis with plates and screws. Histologically, a moderately cellular spindled mesenchymal tumor was observed with focal bone lytic areas and ulceration of the overlying oral mucosa (Figure 2A,B).

**Figure 3:**
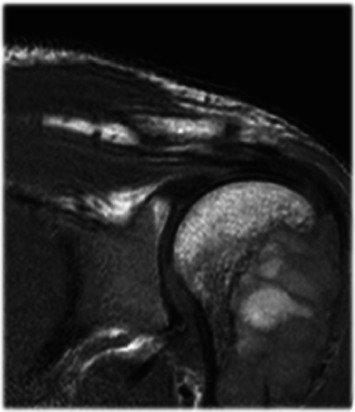
MRI of left shoulder. It shows cyst-like lesion in the proximal humeral area involving the humeral trochitis.

The immunoprofile of the neoplastic cells revealed a weak reactivity for smooth muscle actin, a Ki67 proliferative index of 1%, being stains for desmin, calponin, beta-catenin, and CD34 all negative. The above-described findings support a diagnosis of desmoplastic fibroma (DF) of bone. The patient presented with shoulder pain and difficulty in raising the left arm. On physical examination, a painful swelling was present. A left shoulder magnetic resonance imaging (MRI) was then performed with evidence of a pluri-lobulated cyst-like lesion in the proximal humeral area (6 cm × 3.5 cm × 3 cm) (Figure 3).

The histological evaluation revealed a BT. Subsequent tests highlighted hypercalcemia and hypophosphatemia; this prompted an endocrine referral for the evaluation of suspected PHPT. The patient was unaware of specific hereditary diseases in his family, and he had an unremarkable medical history and was not on medication.

## 3. Diagnostic Assessment

**Figure 4:**
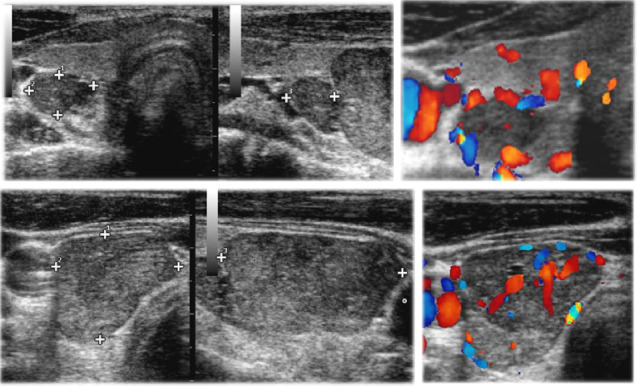
US of neck. It shows two right enlarged parathyroid glands.

**Figure 5:**
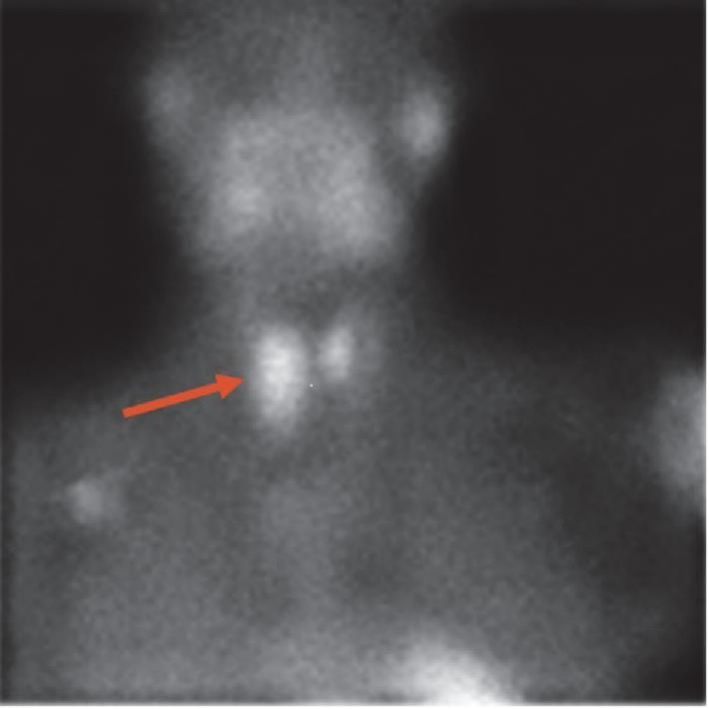
Parathyroid SESTAMIBI scan. It shows two right enlarged parathyroid glands.

The biochemical profile of the patient is reported in Table 1. Ultrasound scan (US) of the neck revealed two lesions behind the lower pole of the right thyroid lobe, hypoechoic, rounded, with regular margins (5.9 mm × 9.6 mm × 7.1 mm and 15.9 mm × 18.5 mm × 27.4 mm, respectively), both consistent with enlarged parathyroid glands (Figure 4). The parathyroid SESTAMIBI scan was consistent with the ultrasound findings (Figure 5). The DXA scan highlighted a *Z*-score <−3SD at the forearm and lumbar spine; no vertebral fractures were detected. The blood exams and the clinical history allowed to rule out the MEN syndromes. Next-generation sequencing (NGS) gene panel evaluated the following genes: AIP, BAP1, CDC73, CDKN1A-1B-2B-2C, DLST, DNMT3A, FH, FLCN, MAX, MDH2, MEN1, PRKAR1A, RET, SDHA-B-C-D-AF2, SLC24A11,TMEM127, TP53, and VHL. A heterozygous CDC73 pathogenic variant c.96>A p.Trp32Ter was identified. No family members were available to extend genetic tests.

## 4. Treatment

A simultaneous right hemithyroidectomy and double right parathyroidectomies (PTX) were performed. Two parathyroid adenomas were histologically reported. The intervention was extended to the thyroid lobe due to the higher risk of parathyroid carcinoma in young patients with HPT-JT syndrome: in this case, the high PTH and calcium levels and the parathyroid size supported the clinical suspicion of PC.

## 5. Outcome and Follow-Up

At the 6 and 12 months postoperative evaluations, the patient was asymptomatic with adequate calcium and vitamin D blood exams, during integrative treatment.

## 6. Discussion

We describe a case of HPT-JT syndrome related to a CDC73 pathogenic variant, associated with a BT of the arm and a rare nonossifying fibroma of the mandible. HPT-JT syndrome is an autosomal dominant disorder associated with inactivation of the CDC73 gene, which encodes for the parafibromin [3]. The most common manifestation of HPT-JT syndrome is PHPT, and over 75% of patients with PC show inactivating CDC73 somatic or germline mutation [4, 5] with consequent reduced or absent parafibromin immunoreactivity. In HPT-JT cases, there is a high incidence of PC, with a risk as high as 15%–20% [6].

HPT-JT syndrome includes non-endocrine neoplasms unrelated to PHPT as bone, renal, and uterine tumors [3]. The jaw tumors occur in up to 50% of cases of HPT-JT [7]. Most tumors in HPT-JT syndrome are ossifying fibromas of the mandible or maxilla and may reach impressive sizes. The management of the ossifying fibromas involves surgical resection, followed by reconstruction in some cases [8, 9]. Surprisingly, in our patient a nonossifying fibroma of the mandible, namely a DF, was identified. It is a benign bone lesion, locally aggressive with infiltrative characteristics. In particular, DF is one of the rarest bone disease [10], generally occurring in childhood. It has an F:M ratio of 2.25:1 and a mean size of 40 mm. DF can affect any bone but the mandible is the most frequently involved site [11]. It is hystologically composed of spindle-shaped cells with minimal cytological atypia and abundant collagen production [12]. Despite its aggressive behavior, no malignant transformation has been reported [13]. Radiographically, DF appears as an osteolytic lesion with frequent extension in the surrounding soft tissues [14].

To date no association of DF, neither with PHPT nor other endocrine diseases, has been described. BT, the classic skeletal manifestation of PHPT, is characterized by subperiosteal bone resorption, often painless with slow and silent evolution. They can be locally destructive and lead to bone fractures and deformity [15]. They may occur elsewhere in PHPT [16], with a prevalence of 0.1% in jaws [17, 18]. BT within long and flat bones in patients with HPT-JT syndrome has been reported [19] and also a case of a BT localized in the maxilla has been described [20]. BT can mimic other lesions, such as fibrous dysplasia (FD), giant cell granuloma (GCG), giant cell tumor (GCT), aneurysmal bone cyst, and cancer metastases [21]. To date, some case reports of association of sporadic PHPT and FD [21, 22], GCG [23], or Aneurysmal Bone Cyst [24] have been reported; however, no association with the HPT-JT has been described. In PHPT-related bone disease, PTX usually results in improved bone mineral density, reduced risk of fracture, and regression of BT [25]. For this reason, the surgical treatment of BT is usually not indicated [26]. For ossifying tumors of the jaw in HPT-JT syndrome, the surgical resection represents the standard treatment.

## 7. Conclusions

In summary, the diagnosis of HPT-JT syndrome-associated bone lesions is challenging, but it is crucial in order to avoid unnecessary surgeries and treatments. The lack of family history has been an additional difficulty and diagnostic challenge in our clinical case.

## Figures and Tables

**Table 1:** Laboratory findings at diagnosis (before parathyroidectomy).

Biochemistry	Test	Reference range
PTH (pg/mL)	702	6.5–36.8
Serum calcium (mg/dL)	12.9	8.7–10.4
Ionized calcium (mMol/L)	1.78	1.15–1.29
Creatinine (mg/dL)	0.66	0.50–1.20
Serum phosphorus (mg/dL)	1.7	2.4–5.1
Serum magnesium (mg/dL)	1.8	1.3–2.7
Alkaline phosphatase (IU/L)	314	43–115
25OHD (ng/mL)	5.9	>30
24-h urine calcium (mg/24 h)	320	110–300

## Data Availability

The data used and analyzed during the current study are available from the corresponding author upon reasonable request.
